# Calmodulin Interaction with hEAG1 Visualized by FRET Microscopy

**DOI:** 10.1371/journal.pone.0010873

**Published:** 2010-05-27

**Authors:** J. Tiago Gonçalves, Walter Stühmer

**Affiliations:** 1 Molecular Biology of Neuronal Signals, Max-Planck Institute for Experimental Medicine, Göttingen, Germany; 2 DFG Research Center for Molecular Physiology of the Brain, Göttingen, Germany; University of California, United States of America

## Abstract

**Background:**

Ca^2+^-mediated regulation of ion channels provides a link between intracellular signaling pathways and membrane electrical activity. Intracellular Ca^2+^ inhibits the voltage-gated potassium channel EAG1 through the direct binding of calmodulin (CaM). Three CaM binding sites (BD-C1: 674-683, BD-C2: 711-721, BD-N: 151-165) have been identified in a peptide screen and were proposed to mediate binding. The participation of the three sites in CaM binding to the native channel, however, remains unclear.

**Methodology/Principal Findings:**

Here we studied the binding of Ca^2+^/CaM to the EAG channel by visualizing the interaction between YFP-labeled CaM and Cerulean-labeled hEAG1 in mammalian cells by FRET. The results of our cellular approach substantiate that two CaM binding sites are predominantly involved; the high-affinity 1-8-14 based CaM binding domain in the N-terminus and the second C-terminal binding domain BD-C2. Mutations at these sites completely abolished CaM binding to hEAG1.

**Conclusions/Significance:**

We demonstrated that the BD-N and BD-C2 binding domains are sufficient for CaM binding to the native channel, and, therefore, that BD-C1 is unable to bind CaM independently.

## Introduction

*Ether-à-go-go* (EAG) channels mediate inward-rectifying potassium currents with activation close to the membrane resting potential. They are reported to be modulated by intracellular Ca^2+^ levels [1], [2], a feature that is likely to play a role in their proposed functions in cell proliferation [3], differentiation (myoblast fusion) [4] and regulation of neuronal activity [5], [6], [7].

EAG channels are the founding members of the the KCNH or ether-a-go-go related family of voltage-gated K^+^ channels [8]. EAG-family channels are structurally characterized by the presence of the highly conserved EAG or PAS (Per-Arnt-Sim) domain in their N-termini [9]. Their currents, however, can differ significantly from those of EAG1 or EAG2. Functional EAG channels are tetramers and both hEAG1 and hEAG2 have been shown to form functional heterotetramers upon co-expression [10]. EAG channel subunits are multipass membrane proteins with six trans-membrane segments (S1-S6) and intracellular tails at the N- and C-termini, a standard topology which is shared by other voltage-gated K^+^ channels [11]. The cytoplasmic regions at the N- and C-termini of the channel comprise over 70% of the aminoacid sequence and contain several functional domains. The N-terminus includes a CaM binding domain (BD-N) and the PAS domain. The latter appears to play a role in channel gating by binding to the S4-S5 intracellular loop [9], [12]. The cytoplasmic C-terminus region contains four regulatory domains- a putative, and apparently non-functional, cyclic nucleotide (cNMP) binding domain [13], [14], two CaM binding domains (BD-C1 and BD-C2) and a tetramerization-mediating domain [15], [16].

hEAG1 was found to be inhibited by Ca^2+^, revealing a half-maximal inhibition at a Ca^2+^ concentration of ∼100 nM [1], [2]. This implies that EAG channel activity is inhibited just above resting Ca^2+^-levels. Initially, Schönherr et al. [17] reported that the inhibition of hEAG1 by Ca^2+^ was mediated by the direct binding of Ca^2+^/CaM to the C-terminus (AA 707–726) of the channel. This stretch of residues harbors a putative amphiphilic CaM-binding helix (BD-C2: 711–720). More recently, two further putative CaM binding domains were found [18] using a peptide scanning approach, one in the N- (BD-N: AA 151–165) and another in the C-terminus (BD-C1: AA 674–683) of hEAG1. Mutations in each of these domains were reported to impair the inhibition of hEAG1 currents by Ca^2+^. Little is presently known about the contribution of BD-C1 to the binding of CaM. Fluorescence Correlation Spectroscopy (FCS) affinity assays indicated that CaM binding to this site is of lower affinity as compared to the other sites [18], raising doubts on whether it actually binds CaM or merely contributes to CaM binding to BD-C2. This kind of ambiguity in the results of biochemical assays for ion-channel interacting proteins is not uncommon. Usually, only the cytosolic portions of channels are assayed and these may not acquire the conformation of the native tetrameric membrane-inserted channel. For these reasons, and in contrast to previous screens, we developed an assay based on Förster Resonance Energy Transfer (FRET) to investigate the binding of CaM to the full-length hEAG1 channel in cells, directly. We show that BD-C1 is not able to bind CaM to the channel on its own and that binding to the N-terminus and the C-terminal BD-C2 suffices.

## Methods

### Plasmid construction and mutagenesis

The C-terminal Cerulean- and YFP-fusion constructs of full-length hEAG1 were created by cloning the cDNA of hEAG1 into the CB6 vector using KpnI and NotI restriction sites. Cerulean and YFP were cloned at the C-terminal of hEAG1 by PCR using NotI and BamHI sites. The Cerulean- and YFP- fusion constructs of the C-terminus of hEAG1 were cloned by amplifying the segment M478-S962 of hEAG1 by PCR and inserting it between the BamHI and NotI sites of the pcDNA3 vector (Invitrogen). Cerulean and YFP were then amplified by PCR with flanking NotI sites and inserted downstream of the C-terminus of hEAG1.

The Glutathione S-transferase (GST)-fusion construct of the N-terminus of rEAG1 was obtained by PCR amplification of the fragment AA 1–219 and subsequent subcloning between BamHI and NotI restriction sites in the pGEX-4T-1 vector (Pharmacia/GE Healthcare).

The cDNA for rat calmodulin II was amplified by PCR with flanking NotI restriction sites and cloned into the CB6-N-YFP vector to generate N-terminally YFP-labeled calmodulin (YFP-CaM). Point mutations in the CaM binding sites of rat and human EAG1 were generated using the PCR-based QuickChange XL mutagenesis kit (Stratagene). All constructs were verified by sequencing.

Cerulean was kindly provided by D. Piston, Vanderbilt University, Nashville, TN, USA, the YFP-CB6 vector[19] was a kind gift from M. Way, Cancer Research UK, London, UK, the cDNA for rat calmodulin II was kindly provided by T M. Shea, University of Iowa, Iowa City, IA, USA and the YFP-apo-Calmodulin (YFP-apoCaM [20]) was a gift from D. Yue, Johns Hopkins University, Baltimore, MD, USA. Mutations in all EF-hand domains render this version of CaM insensitive to Ca^2+^.

### Cell culture and transfection

HEK-293 cells were cultured in DMEM: F12 (1∶1) (Gibco), supplemented with 10% Fetal Calf Serum and 0.1% streptomycin/penicillin (Gibco) in a humidified atmosphere with 5% CO_2_ at 37°C. Cells were seeded on poly-L-lysine-coated glass coverslips and transfected at 20–30% confluency using FuGene6 (Roche), according to the instructions of the manufacturer. The total amount of cDNA used was 0.5 µg per 15 mm cover slip. For co-transfections, a hEAG1:CaM cDNA ratio of 3∶1 (m/m) was used.

### Ca^2+^-ionophore treatment and sample preparation

For FRET measurements on CaM binding, cells were treated 36 hrs after transfection with 1 µM ionomycin (Calbiochem) in either Ca^2+^-containing Ringer solution (145 mM NaCl, 25 mM HEPES pH 7.4, 5.4 mM KCl, 5 mM Glucose, 1.8 mM CaCl_2_, 1 mM NaH_2_PO_4_, 0.8 mM MgSO_4_ - denoted “+Ca^2+^”) or Ca^2+^- and Mg^2+^ -free Ringer solution with 1 mM EGTA (denoted “-Ca^2+^”), for 10 min at 37°C and subsequently fixed for 25 min with 4% formaldehyde. After fixation, cells were briefly washed in PBS and mounted in Mowiol.

### Microscopy

Cells were imaged on a Leica DMIRE2 inverted microscope equipped with a TCS SP2 (AOBS) confocal scanner and a 63x NA1.4 HCX PL Apo objective (Leica). Fluorescence Resonance Energy Transfer (FRET) was measured by Acceptor Photobleaching [21]. Cerulean and YFP were excited using the 458 and 514 nm lines of an Argon laser, respectively. Cerulean emission was recorded in the spectral window of 470–505 nm and YFP at 525–610 nm. The use of 458 nm for Cerulean excitation avoids photoconversion effects of YFP [22]. For FRET efficiency measurements, images of the donor (Cerulean) and acceptor (YFP) were acquired before and after photo-destruction of YFP to 15% of its initial intensity in a selected region of interest. Image size was 1024×1024 pixels and each line was averaged over two consecutive line-scans. The unbleached areas were used as a control for sample movement or laser power fluctuations. Pixels from these areas were included in our analysis (denoted ‘Control’) as a baseline reference. Photomultiplier gain and offset values, as well as laser intensities were set to maximize linearity and kept constant for the images acquired before and after bleaching of the acceptor fluorophore. The pinhole opening was maximally opened during acquisition (5.2 Airy units).

### Image analysis and quantification

Image analysis was performed with the Matlab 7.0 suite (The Mathworks) using custom-written scripts. In brief, donor images were binned (2×2) and subsequently subjected to a low-pass Wiener and Gaussian filter with 3×3 kernel dimensions. Images were background-subtracted and thresholded on fluorescence intensity. FRET efficiencies (E) were calculated on a pixel-to-pixel basis by determining the difference between donor intensities before (Dpre) and after photobleaching (Dpost) of the acceptor and normalizing to the donor intensity after photobleaching (E = 1-(Dpre/Dpost)). The FRET efficiencies are represented in pseudo-color for better visualization. Cumulative FRET efficiency distribution histograms for each experimental condition were obtained by normalization of the cumulative FRET efficiencies per pixel of several cells to the number of pixels analyzed in regions of interest (or unbleached control areas) and the number of cells for each condition. These distributions are probability density functions (PDF), with an integral of unity. Statistical analysis was performed using the Student t-test.

### Overlay assays

Expression of the GST- rEAG1 N-terminus was induced in BL21 (DE3) E. Coli (Stratagene) with 1 mM IPTG. Cells were harvested after 4 hrs, lysed by short sonication and boiled for 5 min in Laemmli's loading buffer. Proteins were separated by SDS-PAGE and either stained with Coomassie brilliant dye or transferred onto nitrocellulose membranes (GE Healthcare). The membranes were blocked overnight at 4°C with 5% (w/v) milk powder in TBS-T (200 mM Tris-HCl pH 7.4, 140 mM NaCl, 0.1% Tween-20), followed by 2 hrs incubation at room temperature with 0.5 µg/ml biotinylated CaM (Calbiochem) and 1 mM CaCl_2_ in TBS-T, washed once for 10 min in 0.5% Triton TX-100, 1 mM CaCl_2_ in TBS-T and twice for 10 min in 1 mM CaCl_2_ in TBS-T. Membranes were then incubated for 1 hr with 0.2 µg/ml HRP-conjugated Streptavidin (Pierce) in TBS-T with 1 mM CaCl_2_, washed four times for 5 min in the same buffer and detected using the Enhanced Chemo-Luminescence kit (ECL, GE Healthcare).

## Results

### A cellular FRET assay for the Ca^2+^-dependent interaction of hEAG1 with CaM

**Figure 1:**
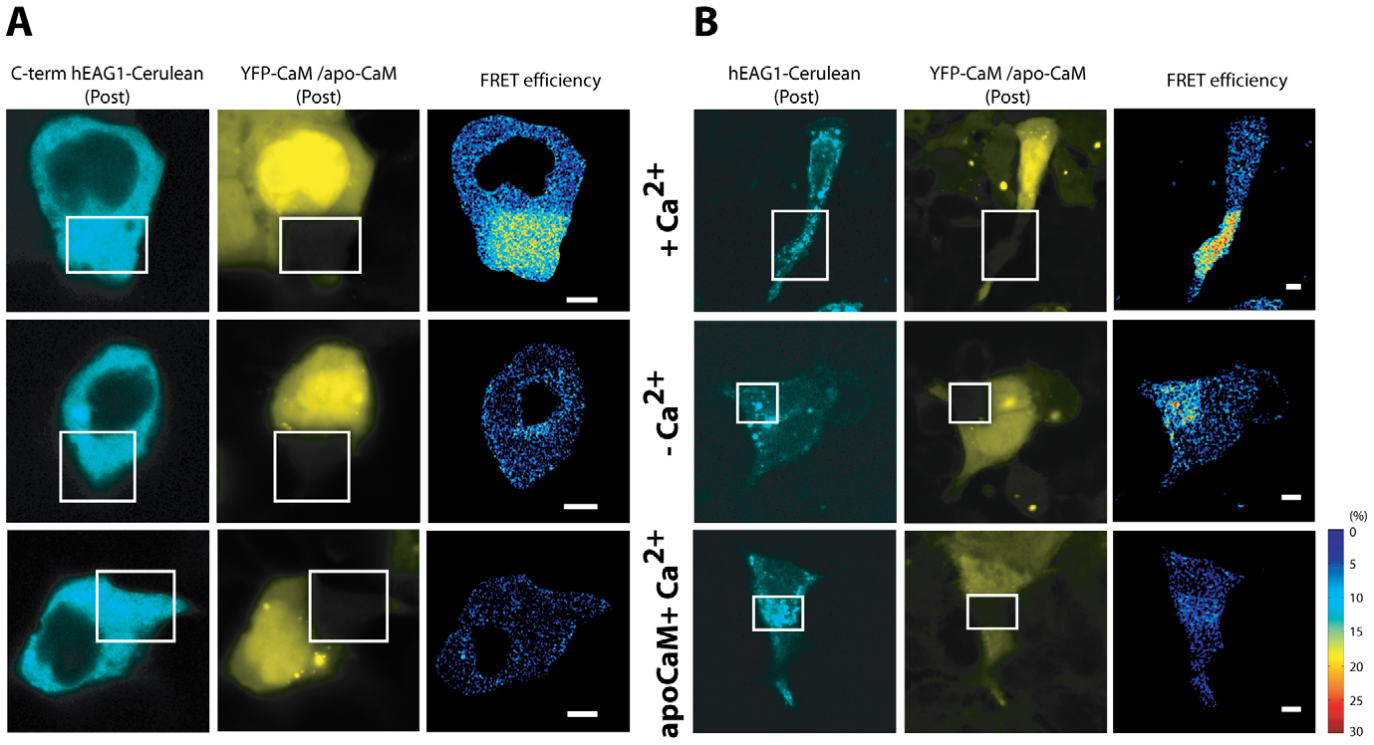
Visualization of the Ca^2+^-dependent interaction of hEAG1-Cerulean with CaM in cells by FRET. Fluorescence intensity and FRET efficiency (E%) images of HEK-293 transfected with (A) the C-terminus of hEAG1 or (B) the full-length hEAG1 labeled with Cerulean (donor) and CaM or apoCaM labeled with YFP (acceptor). Cells were stimulated with 1 µM ionomycin in the presence of 1.8 mM Ca^2+^ (+Ca^2+^) or 1 mM EGTA (-Ca^2+^) for 10 min. FRET was measured by acceptor photobleaching. Bleached areas are indicated by white boxes. FRET efficiencies are shown in pseudo-color. Scale bars: 5 µm.

In order to establish a FRET assay for the Ca^2+^/CaM-mediated regulation of hEAG1 in mammalian cells, channel subunits were fused at their C-terminus to either Cerulean or YFP. The C-terminal labeling of the channel subunits with fluorescent proteins (FPs) did not interfere with the intracellular localization and functionality of the channel. When hEAG1 was fused at the C-terminus with FPs and expressed in several mammalian cell lines, its fluorescence signal was located mostly in small vesicles in the cytoplasm and in the endoplasmic reticulum as well as the connecting nuclear envelope (Fig 1A). Occasionally, a plasma membrane staining was discernible as a faint contour line. This expression pattern was the same as that of non-labelled overexpressed channels as detected by monoclonal antibodies recognizing the intracellular part of the channel. The currents and kinetics of GFP-fused hEAG1 were furthermore indistinguishable from those of non-labeled channels (data not shown).

In order to characterize the interaction of EAG1 with CaM by FRET, the truncated cytosolic C-terminus (AA 478–962) was initially co-expressed with YFP-CaM in HEK cells. The C-terminus of the channel localized uniformly in the cytoplasm and was absent from the nucleus, whereas CaM was present in both these cellular compartments. Optimization experiments showed that FRET between hEAG1 and CaM could only be measured when the channel units were labeled at their C-termini with the donor fluorophore Cerulean and CaM with the acceptor YFP at its N-terminus, and not vice-versa (data not shown). FRET measurements with the acceptor photobleaching method require a molecular excess of the acceptor fluorophore [23], a condition which was probably not met when CaM was labelled with the donor. When hEAG1 was labelled with Cerulean, FRET efficiencies did not depend on the ratio of Cerulean to YFP fluorescence intensities (See File S1 and Fig. S1), suggesting that under these experimental conditions there is an excess of the acceptor fluorophore.

**Figure 2:**
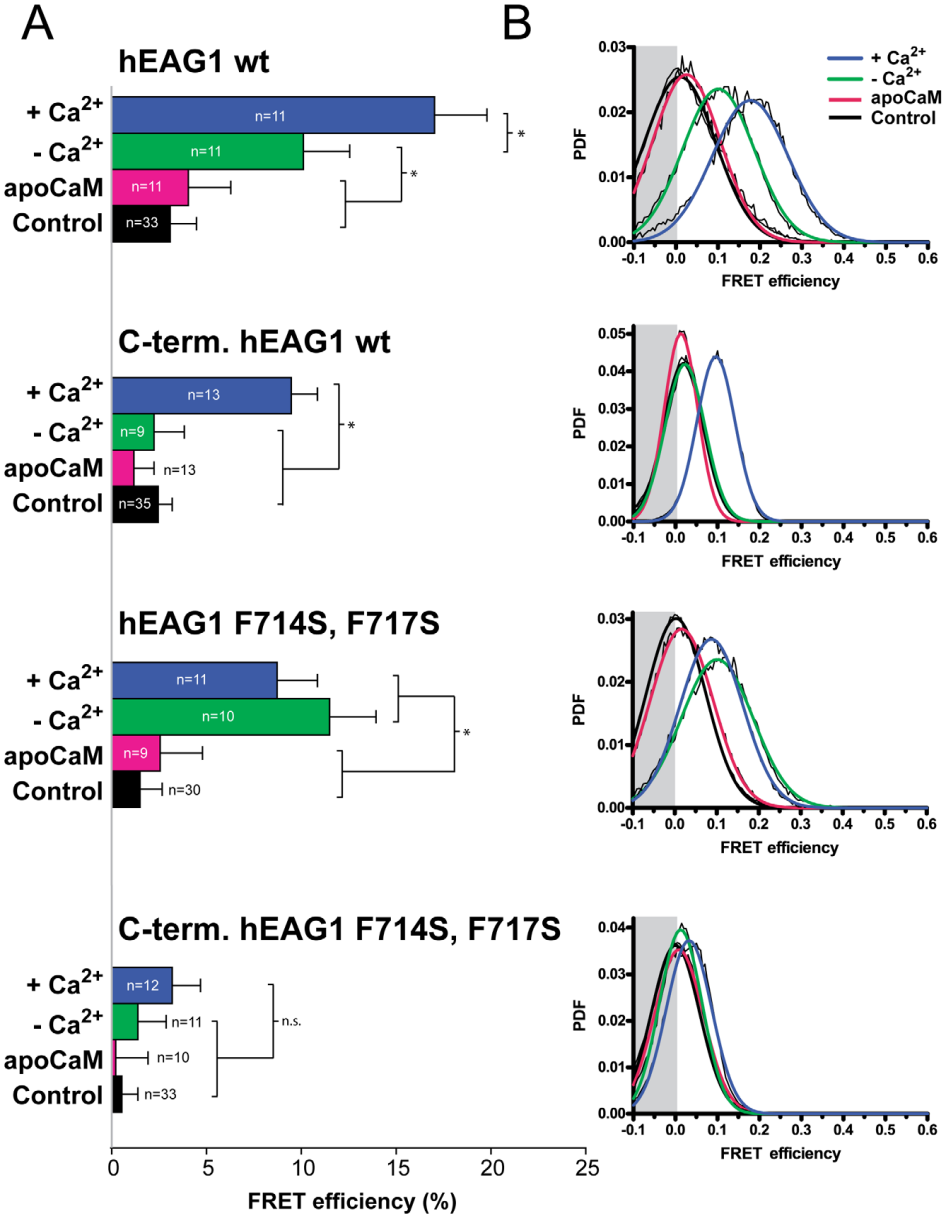
Mutation of BD-C2 (F714S, F717S) disrupts the binding of CaM to the truncated C-terminus of hEAG1 but not to the full channel. The FRET efficiencies of Cerulean-labeled wt-hEAG1 C-terminus and full-length channel upon interaction with YFP-CaM in HEK-293 were determined by acceptor photobleaching and compared to those of hEAG1 with mutated BD-C2 (F714S, F717S) (A) The average FRET efficiencies (± SEM) and (B) Cumulative FRET distribution histograms (probability density function, PDF) of several cells as determined under Ca^2+^-rich and depleted conditions.

To investigate the Ca^2+^-dependency of the hEAG1-CaM interaction, cells were placed in Ca^2+^-containing (1.8 mM Ca^2+^) or Ca^2+^-free Ringer solution (1 mM EGTA) and stimulated with 1 µM ionomycin. Cells were fixed and FRET was measured by the method of acceptor photobleaching (Fig. 1). No differences in the localization of the constructs were seen upon increasing or depleting Ca^2+^. The FRET measurement revealed that the C-terminus of the channel interacts with CaM in a Ca^2+^-dependent manner (Fig. 1 A). Cells with increased Ca^2+^ concentrations showed mean FRET efficiencies of 9.5% (±1.3% SEM, n = 13), whereas negligible FRET was measured (2.2±1.6%, n = 9) in Ca^2+^-depleted cells (p>0.05). In the cumulative FRET efficiency distributions of all cells, this is represented by a shift of the FRET distribution towards zero (Fig. 2 B). This indicates that the C-terminus of hEAG1 only binds CaM in its Ca^2+^-bound state. This was verified by the use of Ca^2+^-insensitive CaM, YFP-apoCaM, as acceptor. Expression levels of YFP-apoCaM in transfected cells were similar to those of YFP-CaM (Fig. S2). The FRET efficiency distribution of apoCaM-co-transfected cells overlaps that of Ca^2+^-depleted cells (1.2±1.0%, n = 13), peaking around zero.

A Ca^2+^-dependent interaction with CaM was also found for the full-length hEAG1 channel (Fig. 1). The channel displayed high FRET efficiencies with YFP-CaM (17.0±2.8%, n = 11) in the presence of Ca^2+^. In Ca^2+^-depleted cells, significantly reduced FRET efficiencies (10.1±2.5%, n = 11, p<0.05) were obtained. Upon co-transfection with YFP-apoCaM, FRET efficiencies were even reduced further (4.0±2.3%, n = 11), even in the presence of Ca^2+^. The occurrence of detectable FRET even after depletion of Ca^2+^ suggests the presence of minimal amounts of Ca^2+^-bound CaM molecules that mediate the binding to the full-length channel. Moreover, it indicates that the full-length hEAG1 channel has a higher affinity for Ca^2+^/CaM than the truncated C- terminus alone.

### Mutation of the BD-C2 (F714S, F717S) prevents binding of CaM to the C-terminus but not to the full-length hEAG1 channel

In order to further investigate the binding of CaM to the C-terminus, of hEAG1, we introduced the mutations F714S, F717S in BD-C2 in the truncated C-terminus and full-length construct, respectively. The expression pattern of the mutated constructs was not changed in comparison to their wild-type counterparts. Mutation of BD-C2 fully inhibited the interaction of CaM with the C-terminus of hEAG1, since no FRET was measured (p>0.05) under both the high (3.2±1.5%, n = 12) and low Ca^2+^ conditions (1.4±1.5%, n = 11), as well as with apoCaM (0.2±1.7%, n = 10) (Fig. 2).

However, the full-length channel containing these mutations interacted with YFP-CaM in the presence of Ca^2+^ (8.7±2.2%, n = 11), as well as upon Ca^2+^ depletion (11.5±2.4%, n = 10). The FRET efficiencies of cells with and without Ca^2+^ were not significantly different. Furthermore, FRET between the BD-C2 mutated channel constructs and YFP-apoCaM was negligible (2.6±2.2%, n = 9), verifying that the interaction is not due to binding of Ca^2+^-unbound CaM. These results show that the previously reported mutations in BD-C2 (F714S, F717S) fully prevent the binding of CaM to the truncated, cytosolic C-terminus of the hEAG1 and that the intact BD-C1 alone is not sufficient for CaM binding. However, the mutated full-length protein is still able to bind CaM, either through BD-N or a combination of BD-N and BD-C1.

### Mutations F151S, A152S prevent the binding of CaM to the N-terminus of EAG1

**Figure 3:**
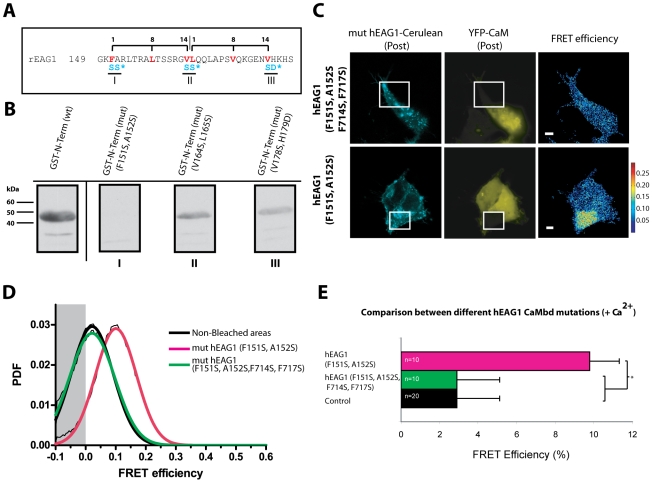
Mutation of both BD-N and BD-C2 completely disrupt the binding of CaM to full-length hEAG1. (A) Amino acid sequence of AA 147-209 of EAG1 with the two putative 1-8-14 CaM binding motifs highlighted. (B) CaM overlay blot of the 1-8-14 mutated N-terminus of rEAG1. Detection with HRP-conjugated streptavidin revealed that mutations F151S, A152S (I) completely disrupted binding of biotinylated CaM whereas V164S, L165S (II) and V178S, H179D (III) only caused a reduction in binding. (C–E) FRET measurements in cells transfected with YFP-CaM and Cerulean-labeled full-length hEAG1, mutated in BD-N (F151S, A152S) or in both BD-N and BD-C2 (F151S, A152S, F714S, F717S). Cells were treated with 1 µM ionomycin in the presence of high Ca^2+^. (C) Fluorescence intensity images of Cerulean-hEAG and YFP-CaM after photobleaching and the corresponding FRET efficiency images in pseudo-color (D) Cumulative FRET distribution histograms (probability density function, PDF) and (E) average FRET efficiencies (± SEM) of several cells. Scale bar: 5 µm.

Upon closer inspection of the sequence of the N-terminus, two adjacent putative Ca^2+^-dependent CaM-binding motifs of the type “1-8-14” [24] were found at AA 151–164 and 165–178 (Fig. 3 A). Both sequences are located in a stretch that is predicted to be amphipathic and composed mostly of basic and hydrophobic residues. The second putative binding motif contains a proline, an amino acid that seldom appears in amphipathic helices, although this does not imply that this site cannot be involved in CaM binding.

Mutations affecting both 1-8-14 motifs were introduced in a GST-fusion of the N-terminal segment AA 147–209 of rEAG1 in order to localize the residues needed for the interaction with CaM and to determine if both motifs are fully functional. CaM binding sites of the 1-8-14 type are particularly sensitive to mutations in the first (position 1) and last (position 14) hydrophobic residues [24]. Three pairs of mutations were inserted. The mutations F151S and A152S were aimed at disrupting the hydrophobic region at the beginning of the first 1-8-14 motif (AA 151–165). They should only affect this first motif and therefore provide information on whether both or only the first of the putative motifs actively binds CaM. The middle mutations, V164S and L165S, were introduced with the intent of disrupting both adjacent 1-8-14 motifs in one construct. The third pair of mutations - V178S, H179D - target the last hydrophobic residue of the second putative 1-8-14 binding site (AA 165–178).

The three constructs thus obtained were expressed in bacteria and tested biochemically for CaM binding on an overlay blot assay (Fig. 3 B). Mutants V164S, L165S and V178S, H179D, involving the second 1-8-14 motif, display a reduced binding to biotinylated CaM, in comparison to the wild-type GST-N-terminus However, only the mutation pair F151S, A152S, at the beginning of the first 1-8-14 motif, led to the complete disruption of binding. These results show that the first 1-8-14 binding motif is the dominant CaM-binding domain in the N-terminus cytosolic segment of EAG1.

### hEAG1 channels with disrupted BD-N, BD-C2 and intact BD-C1 are unable to bind CaM

In order to investigate the effect of BD-N on CaM binding, mutations F151S, A152S were introduced in both wild-type and BD-C2-mutated hEAG1 and FRET was measured by acceptor photobleaching in HEK293 cells (Fig. 3 C).

Fusion constructs of the full-length hEAG1 containing only the BD-N mutations F151S and A152S interacted with YFP-CaM (9.8±2.2%, n = 10) in the presence of Ca^2+^. However, constructs containing both mutated BD-N and BD-C2 were found not to interact with YFP-CaM, as no appreciable FRET (2.9±2.2%, n = 10) could be measured. The combined four mutations F151S, A152S, F714S, F717S are therefore sufficient to hinder the binding of CaM to hEAG1 (p>0.05) BD-C1 and other auxiliary sites can still be involved in the binding mechanism in a cooperative manner. We conclude that these two CaM binding sites, the high-affinity 1-8-14 CaM binding domain in the N-terminus and the previously described C-terminal binding domain at AA 707-276, are the major binding sites involved in CaM binding to EAG1.

## Discussion

hEAG1 channels are known to be inhibited by Ca^2+^/CaM [1], [17], [18]. Based on a peptide screen, three CaM binding sites have been reported, one in the N-terminus (BD-N) and two in the C-terminus (BD-C1 and BD-C1). Although mutations in all three domains have been shown to reduce the inhibition of hEAG1 currents by Ca^2+^, the participation of the three sites in the direct binding of CaM to the native channel remained unclear. Here we describe the use of a FRET-based assay for the characterization of the binding of CaM to voltage-gated potassium channels hEAG1 in mammalian cells. Using our FRET-based assay we were able to show that mutations in both the BD-N and BD-C2 CaM-binding domains were sufficient to fully abolish CaM-binding. This implies that BD-C1 cannot bind CaM on its own and consequently that these two binding sites are the two major sites involved in the direct binding of CaM to native hEAG1 channels.

The relative affinity of the individual CaM binding sites and their contribution to CaM binding to the native EAG1 channel was not entirely clear from previous in vitro studies [18]. The binding affinity for Ca^2+^/CaM to BD-N in FCS and Surface Plasmon Resonance (SPR) studies depended on the length of the peptides used. BD-N was found to have approximately the same binding affinity for Ca^2+^/CaM as the BD-C2 when GST-fusion protein segments were used. However, when the FCS assay was repeated with peptides encoding the minimal CaM-BDs, BD-N was found to have a higher affinity than that of the C-terminus (K_D_ = 45 and 223 nM, respectively). Our data is clearly in favour of the latter situation: the channel with intact BD-N was found to interact with CaM even in Ca^2+^-depleted cells, whereas the C-terminus was found not to bind CaM under the same conditions. This difference probably reflects a higher affinity for Ca^2+^/CaM of BD-N. Typically, 1-8-14-based CaM binding motifs as present in the BD-N are known to have very high binding affinities for Ca^2+^-bound CaM [24].

The results of the peptide screen were also inconclusive about the CaM-binding affinity of BD-C1. A low affinity (Kd>5 uM) was found when using GST-fusion segments, implying that this site is unable to bind CaM independently. On the other hand, the binding affinity was higher (K_D_ = 327 nM) when using fluorescently labeled peptides (∼20 AA). Our studies show that the disruption of BD-N and BD-C2 is sufficient to hinder the binding of CaM to full-length hEAG1, although we can also not exclude the possibility that BD-C1 is involved in CaM binding in a cooperative manner.

The mechanism of CaM inhibition of EAG appears to be substantially different from that of Ca^2+^-activated potassium channels, which have been shown to constitutively bind CaM independently of Ca^2+^ concentration (see [25], [26] for a review). However, two channel families with structural and functional similarities to EAG were also found to contain CaM binding domains, namely Cyclic Nucleotide Gated (CNG) [27], [28], [29] and KCNQ channels [30], [31]. KCNQ channels constitutively bind CaM through a IQ-like CaM binding motif [24], CaM bound to the channel in this manner mediates the inhibition of the channels by Ca^2+^. The mechanism of Ca^2+^/CaM regulation of KCNQ channels is therefore different from that of EAG channels, despite their structural and electrophysiological similarities. CNG channels are the closest structural relatives of the EAG channel family. They share several structural features with EAG including a cyclic-nucleotide gating domain that, although partially conserved from a sequence point of view, was found not to be functional in EAG1[13], [14]. The mechanism of CaM/Ca^2+^ modulation of CNG channels is complex and depends on their composition. Olfactory CNG channels are inhibited primarily by constitutively-bound CaM through IQ-like domains present in CNGA4 and CNGB1b subunits [27], [32], whereas rod CNG channels are possibly inhibited through the binding of Ca^2+^-associated CaM [27], [29]. An IQ-like CaM binding domain in the N-terminus of CNGB1a subunits appears to mediate an intersubunit interaction with the C-terminus of CNGA1 subunit. Ca^2+^/CaM seems to disrupt this thereby inhibiting the channel [29]. We have investigated if a similar inhibition mechanism is present in EAG1, however, no binding of N- and C-terminus was detected on an overlay blot both in the presence and absence of Ca^2+^/CaM (data not shown). In a different approach, we constructed an intramolecular FRET sensor where the full-length EAG1 was labeled with donor and acceptor fluorophores at its cytosolic termini. However, we were unable to measure differences in FRET efficiency with varying Ca^2+^ concentrations (data not shown).

In summary, the results obtained with our FRET assay therefore substantiate the previous model of hEAG1 – CaM interactions and conform to the idea that, under native conditions, disruption of BD-N and BD-C2 is sufficient to impair the binding of CaM to the hEAG1 channel.

## Supporting Information

Figure S1 hEAG1-CaM FRET efficiencies are not influenced by fluorescence intensity. Dependence of FRET efficiencies on the Cerulean/YFP fluorescence intensity ratio (upper panels) and YFP intensity (lower panels), for representative experiments with full-length hEAG1 (A–C) and the truncated C-terminal (D–F). Since PMT gains were optimized for each of the cells imaged, 8-bit pixel intensity values were divided by PMT voltages in order to determine an Adjusted Fluorescence Intensity that can be directly compared between cells. Solid lines represent linear regression fits of the data and dotted lines represent the confidence interval of the best-fit line. A goodness-of-fit (r^2^) figure is indicated in each plot. None of the best-fit lines had a slope significantly different from zero (F-test with zero-slope line as null-hypothesis), indicating that under the experimental conditions used, and for the range of fluorophore concentrations present in the samples, FRET efficiency does not depend on the fluorescence intensities of donor and acceptor fluorophores.
(0.85 MB PDF)

Figure S2 Expression levels of YFP-CaM and YFP-apoCaM are similar, as determined by fluorescence intensity levels. Average fluorescence intensities of cells expressing YFP-CaM or YFP-apoCaM in two representative experiments where both constructs were co-transfected with Cerulean-tagged C-term. hEAG1 (A) or C-term hEAG1 with BD-C2 mutations (B). Since PMT gains were optimized for each of the cells imaged, 8-bit pixel intensity values were divided by PMT voltages in order to determine an Adjusted Fluorescence Intensity that can be directly compared between cells. Laser power was kept constant for each experiment. In experiment A intensity levels of CaM were slightly higher than apoCaM (0.48±0.02 and 0.34±0.02, respectively, p<0.05), however in experiment B intensity levels of both constructs were the same.
(0.18 MB PDF)

File S1 Supporting discussion.
(0.04 MB DOC)
