# Supplementary figures and images for: Calmodulin Interaction with hEAG1 Visualized by FRET Microscopy
Source: PLoS One. 2010 May 27;5(5):e10873. doi: 10.1371/journal.pone.0010873 (PMC2877719; doi:10.1371/journal.pone.0010873)

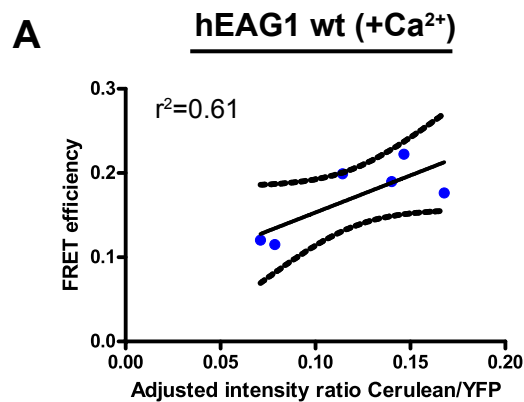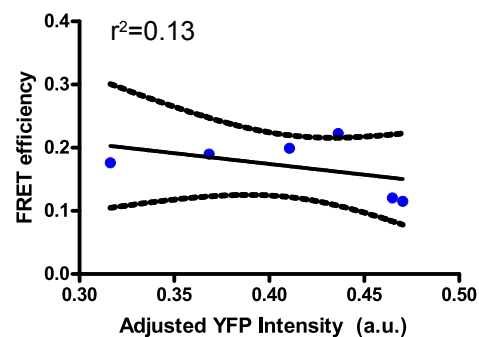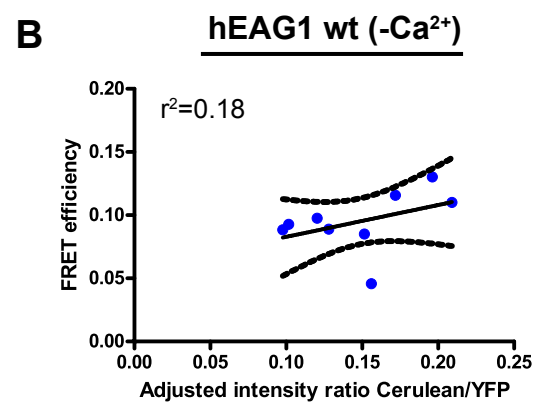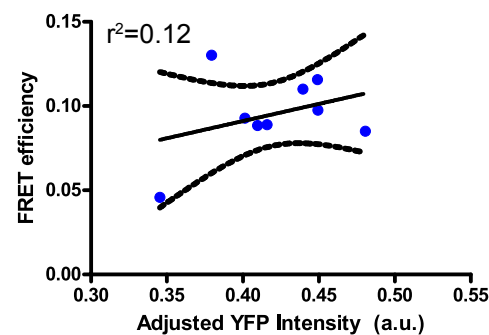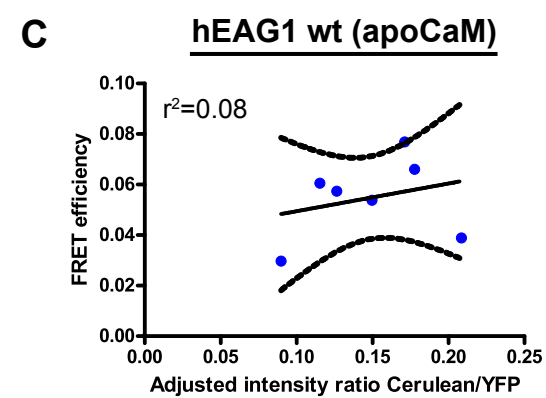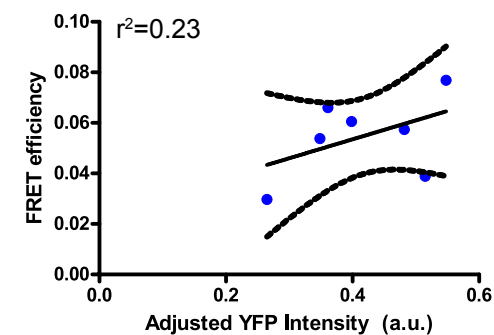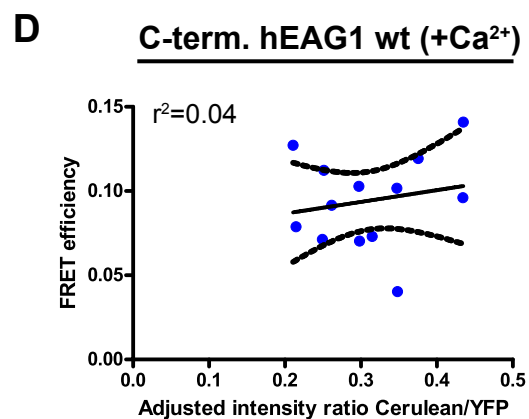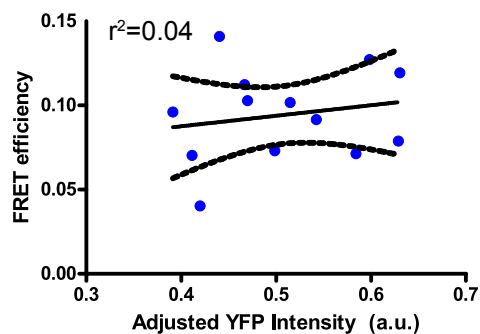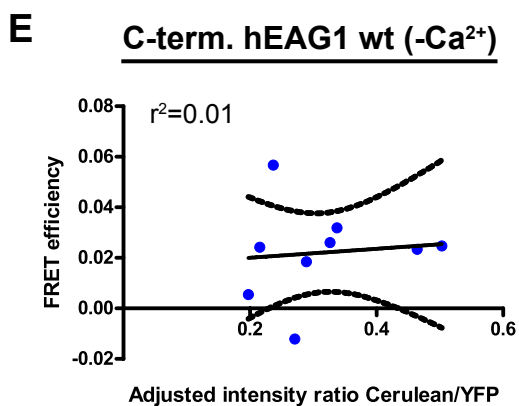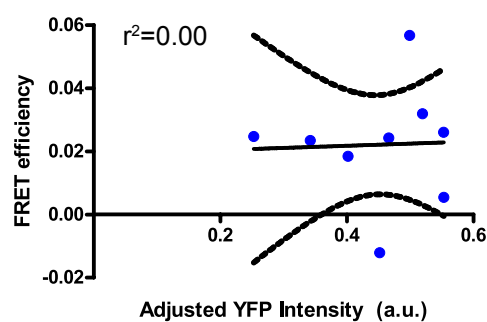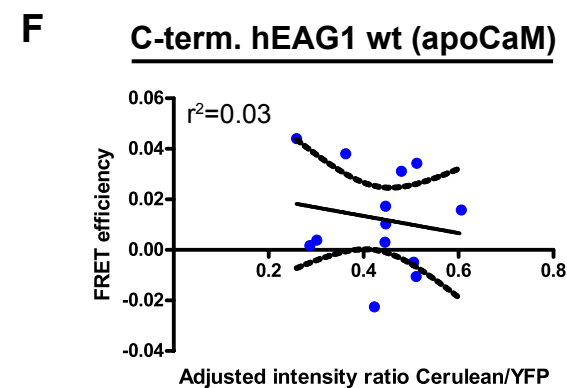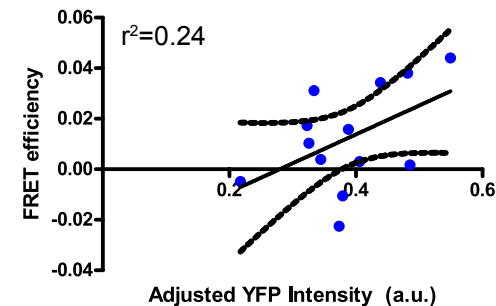

Figure S1

Supplement: Figure S1 — hEAG1-CaM FRET efficiencies are not influenced by fluorescence intensity. Dependence of FRET efficiencies on the Cerulean/YFP fluorescence intensity ratio (upper panels) and YFP intensity (lower panels), for representative experiments with full-length hEAG1 (A–C) and the truncated C-terminal (D–F). Since PMT gains were optimized for each of the cells imaged, 8-bit pixel intensity values were divided by PMT voltages in order to determine an Adjusted Fluorescence Intensity that can be directly compared between cells. Solid lines represent linear regression fits of the data and dotted lines represent the confidence interval of the best-fit line. A goodness-of-fit (r2) figure is indicated in each plot. None of the best-fit lines had a slope significantly different from zero (F-test with zero-slope line as null-hypothesis), indicating that under the experimental conditions used, and for the range of fluorophore concentrations present in the samples, FRET efficiency does not depend on the fluorescence intensities of donor and acceptor fluorophores. (0.85 MB PDF) [file pone.0010873.s001.pdf]

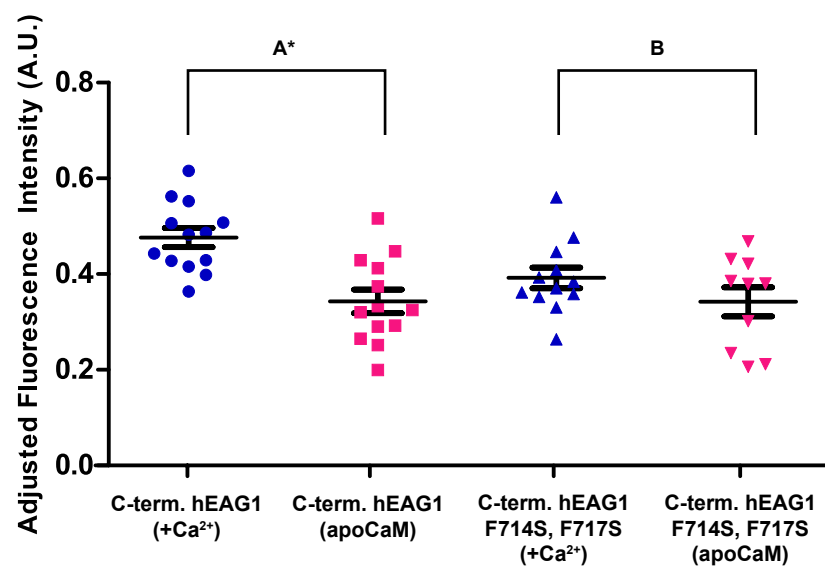

Fig. S2

Supplement: Figure S2 — Expression levels of YFP-CaM and YFP-apoCaM are similar, as determined by fluorescence intensity levels. Average fluorescence intensities of cells expressing YFP-CaM or YFP-apoCaM in two representative experiments where both constructs were co-transfected with Cerulean-tagged C-term. hEAG1 (A) or C-term hEAG1 with BD-C2 mutations (B). Since PMT gains were optimized for each of the cells imaged, 8-bit pixel intensity values were divided by PMT voltages in order to determine an Adjusted Fluorescence Intensity that can be directly compared between cells. Laser power was kept constant for each experiment. In experiment A intensity levels of CaM were slightly higher than apoCaM (0.48±0.02 and 0.34±0.02, respectively, p<0.05), however in experiment B intensity levels of both constructs were the same. (0.18 MB PDF) [file pone.0010873.s002.pdf]
