# Supplementary material for: Calmodulin Interaction with hEAG1 Visualized by FRET Microscopy
Source: PLoS One. 2010 May 27;5(5):e10873. doi: 10.1371/journal.pone.0010873 (PMC2877719; doi:10.1371/journal.pone.0010873)
Supplement: File S1 — Supporting discussion. (0.04 MB DOC) [file pone.0010873.s003.doc]

**Supporting Information for**

**Calmodulin Interaction with hEAG1 visualized by FRET microscopy**

**José Tiago Gonçalves and Walter Stühmer**

**Supporting Discussion**

When the method of acceptor photobleaching is used, measured (or apparent) FRET efficiencies can differ significantly from intrinsic FRET efficiencies if acceptor concentrations are low. Assuming a fixed stoichiometry for a given interaction, FRET efficiencies are expected to depend strongly on the ratio of donor to acceptor concentration, whenever donor molecules outnumber the acceptor. This occurs because a significant proportion of donor molecules will remain uncoupled, thereby diluting the increase in donor fluorescence upon bleaching of the acceptor. For this reason it is important to design FRET experiments so that a molar excess of the acceptor is present. Under these circumstances, FRET efficiencies are not expected to depend on the donor:acceptor ratio, and the measured efficiencies will approach the true, intrinsic FRET efficiency. In order to determine whether in our experiments FRET efficiencies varied with fluorophore concentrations we plotted their dependence on the ratio of Cerulean:YFP. This was done for six representative experiments with both the full-length hEAG1 and the cytosolic C-terminus (Fig. S1). In all cases no significant dependence was found between FRET efficiencies and donor:acceptor ratio (significance determined by linear regression analysis and F-test using a zero-slope line as the null-hypothesis). This result is strongly indicative of the presence of a molar excess of acceptor fluorophore, a condition that must be met for an accurate estimate of intrinsic FRET efficiencies. Experiments using the full-length channel do display a tendency for higher FRET efficiencies at higher donor:acceptor ratios. This tendency can probably be accounted by the punctuate pattern of hEAG1 expression, areas where only a membrane staining is visible are fainter than punctate areas, resulting in noisier FRET measurements. Indeed, the FRET efficiency distributions of full-length constructs are broader than those of the cytosolic C-terminus (see Fig. 2).

Although the presence of a molar excess of the acceptor is desirable for photobleaching FRET experiments, under certain rare circumstances the presence of large amounts of the acceptor fluorophore may cause the occurrence of spurious FRET due to random intermolecular interactions. This may be of particular concern with membrane proteins, since their motional freedom is restricted. In order to confirm that high acceptor concentrations were not causing spurious FRET in our experiments we plotted FRET efficiencies against YFP intensities (Fig. S1) and found no dependence of the former on the latter. Additionally, and because mutant apoCaM is used as a control for binding in the absence of Ca^2+^, we verified that both wild-type and mutant CaM constructs are expressed in the similar amounts (Fig. S2). Altogether, these results confirm that, under these experimental conditions and cellular expression levels, FRET efficiencies are independent from acceptor concentration and donor:acceptor ratio.
